# A common feature pharmacophore for FDA-approved drugs inhibiting the Ebola virus

**DOI:** 10.12688/f1000research.5741.2

**Published:** 2014-12-12

**Authors:** Sean Ekins, Joel S. Freundlich, Megan Coffee

**Affiliations:** 1 Collaborations in Chemistry, Fuquay-Varina, NC, 27526, USA; 2 Collaborative Drug Discovery, Burlingame, CA, 94010, USA; 3 Departments of Pharmacology & Physiology and Medicine, Center for Emerging and Reemerging Pathogens, UMDNJ - New Jersey Medical School, NJ, 07103, USA; 4 Center for Infectious Diseases and Emerging Readiness, University of California, Berkeley, CA, 94720, USA

**Keywords:** ebola virus, computational models, machine learning

## Abstract

We are currently faced with a global infectious disease crisis which has been anticipated for decades. While many promising biotherapeutics are being tested, the search for a small molecule has yet to deliver an approved drug or therapeutic for the Ebola or similar filoviruses that cause haemorrhagic fever. Two recent high throughput screens published in 2013 did however identify several hits that progressed to animal studies that are FDA approved drugs used for other indications. The current computational analysis uses these molecules from two different structural classes to construct a common features pharmacophore. This ligand-based pharmacophore implicates a possible common target or mechanism that could be further explored. A recent structure based design project yielded nine co-crystal structures of pyrrolidinone inhibitors bound to the viral protein 35 (VP35). When receptor-ligand pharmacophores based on the analogs of these molecules and the protein structures were constructed, the molecular features partially overlapped with the common features of solely ligand-based pharmacophore models based on FDA approved drugs. These previously identified FDA approved drugs with activity against Ebola were therefore docked into this protein. The antimalarials chloroquine and amodiaquine docked favorably in VP35. We propose that these drugs identified to date as inhibitors of the Ebola virus may be targeting VP35. These computational models may provide preliminary insights into the molecular features that are responsible for their activity against Ebola virus *in vitro *and *in vivo* and we propose that this hypothesis could be readily tested.

## Introduction

The current Ebola virus (EBOV) crisis has demonstrated that globally we are not prepared to respond with therapeutics to treat existing infections or act as prophylactics as there is no Food and Drug Administration (FDA) or European Medicines Agency (EMEA) approved therapeutic. More importantly this suggests we should have been prepared for a pathogen which has been known about for nearly forty years. The current EBOV outbreak is already proving remarkably costly in terms of the mortality and financial ramifications ^1, 2^. The best approaches to EBOV so far have relied on public health measures for containment ^3^ which have been used in past outbreaks ^4^. These lessons with EBOV will undoubtedly be important for the next virus outbreak ^5^ but they also raise many questions ^6^ which point to how little we know about these viruses in general, as well as how best to share knowledge openly ^7^.

There have been a relatively small number of studies that have attempted to identify compounds active against EBOV. Two recent studies utilized high-throughput screens of a subset of FDA approved drugs against different EBOV strains (Zaire and Sudan) *in vitro* and *in vivo*. These independent reports suggested the promise of the antimalarials amodiaquine and chloroquine in one study ^8^, while the selective estrogen receptor modulators (SERMs) clomiphene and toremifene were active in another ^9^. Chloroquine to date has not progressed beyond the mouse EBOV model used in these studies. We hypothesized that we could use these four molecules to computationally define the features that are important for activity. The previous studies were not exhaustive screens of all FDA drugs and so we have taken this opportunity to suggest additional compounds. Looked at from another perspective “non-antiviral” drugs may be worth following up even though their molecular mechanism is unknown. These compounds may themselves have broad antiviral activity as reports describe modest inhibitory activity against other viruses ^10– 13^.

Several studies have identified non-FDA approved drugs including an *in silico* docking approach to identify molecules targeting the viral Nedd4-PPxY interface ^14^. These molecules were similar to the FDA benzimidazole and aminoquinoline ^8, 9^ compounds that were active against EBOV. Another good example is the recent *in silico* docking of 5.4 million drug-like compounds docked in the viral protein VP35 protein ^15^. This identified multiple pyrrolidinones which inhibit its polymerase cofactor activity ^15^. The pyrrolidinones bind to an alpha helix which is proposed as important for viral function ^16^. With the limited knowledge of small molecules and potential targets we have studied whether the FDA-approved drugs that are active *in vitro* and *in vivo* versus EBOV could be targeting VP35.

## Methods

### Common features pharmacophore for EBOV actives

Two papers from 2013 described compounds active as inhibitors of different EBOV strains *in vitro* and *in vivo*, namely amodiaquine and chloroquine in one study ^8^, clomiphene and toremifene in another ^9^. These active molecules were used as they have both *in vitro* and *in vivo* activity to build a common features pharmacophore with Discovery Studio 4.1 (Biovia, San Diego, CA) from 3D conformations of the molecules generated with the CAESAR algorithm. This identified key features. The pharmacophore was then used to search various databases (for which up to 100 molecule conformations with the FAST conformer generation method with the maximum energy threshold of 20 kcal/mol, were created). The pharmacophore was then used to search the Microsource Spectrum database ( http://www.msdiscovery.com/spectrum.html) as well as the CDD FDA drugs dataset ( https://www.collaborativedrug.com/pages/public_access). In both cases over 300 hits were retrieved initially. The van der Waals surface of amodiaquine (which was more potent than chloroquine ^8^) was added to limit the number of hits retrieved ^17– 19^.

### Receptor-ligand pharmacophores for VP35

Receptor-ligand pharmacophores for the VP35 protein were generated from crystal structures (4IBB, 4IBC, 4IBD, 4IBE, 4IBF, 4IBG, 4IBI, 4IBJ, 4IBK) in the protein data bank PDB. Pharmacophores were constructed using the receptor-ligand pharmacophore generation protocol in Discovery Studio version 4.1 (Biovia, San Diego, CA) with a maximum number of pharmacophores (10), minimum features (4), and maximum number of features (6) as are described elsewhere ^20^.

### *In silico* docking of molecules in VP35 structure

PDB 4IBI was used for docking using LibDock in Discovery Studio (Biovia, San Diego CA) ^21^. The proposed binding site was centered on the ligand and a site sphere created (coordinates 2.14, 20.93, 1.71) with 9.45 Å diameter. The protocol included 10 hotspots and docking tolerance (0.25). The FAST conformation method was also used along with steepest descent minimization with CHARMm. Further parameters followed the default settings. The ligand VPL57 was removed from the binding site and re-docked. The four FDA approved drugs with activity against Ebola were docked in the structure from an sdf file. Molecules were visualized alongside the original ligand VPL57 and the 2D interaction plots generated.

## Results

Pharmacophores, receptor ligand models and docking data for FDA-approved drugs inhibiting the Ebola virus Data was downloaded sourced from Microsource Spectrum and CDD Drugs. Dataset includes sd files used to create the 3D database that was searched. Note that models only run on Discovery Studio.

### Common features pharmacophore for EBOV actives

**Figure 1.**
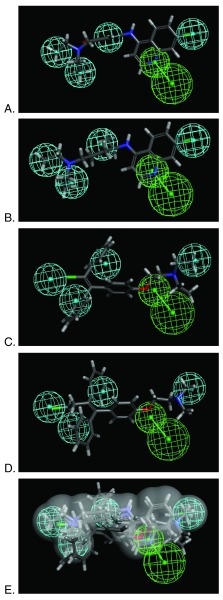
Pharmacophore based on 4 hits. **A**. amodiaquine, **B**. chloroquine, **C**. clomiphene **D**. toremifene and **E**. Overlap showing all molecules in the van der Waals surface of amodiaquine. Pharmacophore features are Hydrophobic (H, cyan) and Hydrogen bond acceptor (HBA, green).

The pharmacophore was generated using the *in vivo* and *in vitro* active amodiaquine, chloroquine, clomiphene and toremifene ( Supplemental Table 1) as these represent the most relevant FDA approved drugs to date. This pharmacophore consists of 4 hydrophobic features and a hydrogen bond acceptor feature ( Figure 1). The pharmacophore with van der Waals surface was also used to search FDA drug various libraries ( Supplemental Table 2 and Supplemental Table 3). The most interesting observations from this virtual screen are that various estradiol analogs score well (e.g. estradiol valerate Fit value 4.23). Previously estradiol was suggested to be active in the EBOV pseudotype assay *in vitro* ^8^. In addition, dibucaine was also retrieved (Fit value 1.58) which was also active in the EBOV pseudotype assay ^8^. Amodiaquine, chloroquine, clomiphene and toremifene can be used as positive controls for future screens. Because the original complete sets of FDA approved compounds screened are not publically accessible it is difficult to compare hit rates versus all compounds tested to date.

### Receptor-ligand pharmacophores for VP35

The nine receptor-ligand pharmacophores created all consisted of three to four hydrophobic features and one to two hydrogen bonding features ( Table 1). Eight of these pharmacophores also had a negative ionizable feature. These suggest that the receptor-ligand based approach results in a general similarity across the nine structures, likely indicating the similar binding mode and importance of features for interfering with this generally hydrophobic pocket for protein-protein interactions.

**Table 1.** Pharmacophores for EBOV VP35 generated from crystal structures in the protein data bank PDB. Pharmacophores were generated using the receptor-ligand pharmacophore generation protocol in Discovery Studio version 4.1 (Biovia, San Diego, CA) with minimum features (3) and maximum features (6). Pharmacophore features are Hydrophobic (H, cyan), Hydrogen bond acceptor (HBA, green), hydrogen bond donor (HBD, purple) and 1 negative ionizable (neg, blue). Excluded volumes (grey) were also automatically added. Further details on this approach are described elsewhere ^20^.

PDB	Pharmacophore features	Pharmacophore with ligand mapped
4IBB	4H, 1HBD, 1 neg ionizable	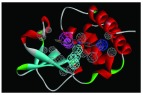
4IBC	3H, 2HBA, 1 neg ionizable	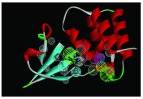
4IBD	4H, 1 HBA, 1 neg ionizable	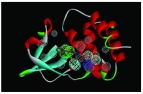
4IBE	4H, 1HBA	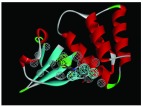
4IBF	4H, 1 HBA, 1 neg ionizable	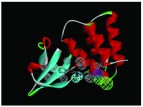
4IBG	3H, 2 HBA, 1 neg ionizable	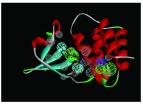
4IBI	4H, 1HBA, 1 neg ionizable	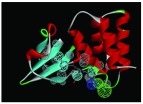
4IBJ	4H, 1HBA, 1 neg ionizable	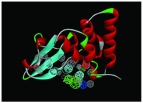
4IBK	4H, 1HBA, 1 neg ionizable	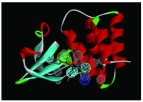

### *In silico* docking of molecules in VP35 structure

**Figure 2.**
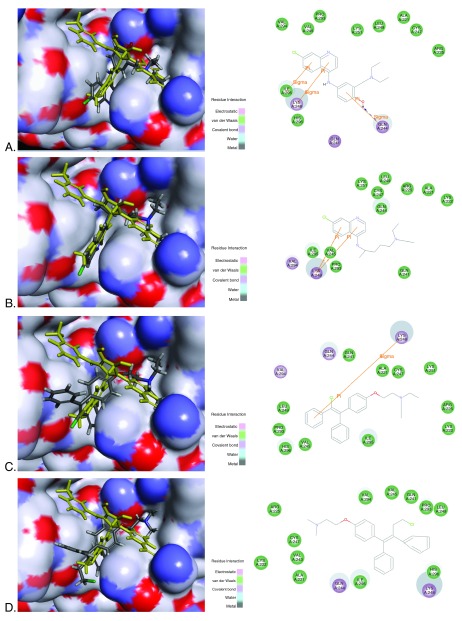
Docking FDA approved compounds in VP35 protein showing overlap with ligand (yellow) and 2D interaction diagram. 4IBI was used, 4IBI ligand VPL57 shown in yellow. **A**. Amodiaquine (grey) and 4IBI LibDock score 90.80, **B**. Chloroquine (grey) LibDock score 97.82, **C**. Clomiphene (grey) and 4IBI LibDock score 69.77, **D**. Toremifene (grey) and 4IBI LibDock score 68.11

**Figure S1.**
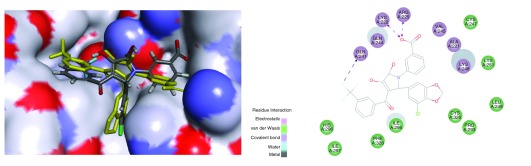
Redocking VPL57 in 4IBI. The 4IBI ligand was removed from the structure and redocked. The closest pose (grey) was ranked 29 with RMSD 3.02A and LibDock score 86.62 when compared to the actual ligand in 4IBI (yellow).

Redocking the 4IBI ligand in the protein resulted in an RMSD of 3.02Å, which generally indicates the difficulty of predicting orientations for compounds binding in what is a relatively hydrophobic and shallow pocket ( Figure S1). This molecule was ranked the 29 ^th^ pose and had a LibDock score of 86.62 ( Figure S1 higher scores are better). The four FDA approved drugs were docked into the VP35 structure 4IBI. All compounds docked similarly and overlapped with the co-crystal ligand ( Figure 2). Amodiaquine and chloroquine had higher LibDock scores (> 90) than the 4IBI ligand, while clomiphene and toremifene had LibDock scores less than 70. All four FDA approved drugs bound similarly to the pyrrolidinone ligands in the pocket formed by residues from the α-helical and β-sheet subdomains ^15^. We have highlighted proposed energetically favorable interactions of the antimalarial candidate binders with ILE295, LYS248 and GLN244, which scored favorably. Previously published studies suggested mutation of ILE295, LYS248 resulted in near-complete loss of binding activity ^15^.

## Discussion

Our previous experience with common feature and quantitative pharmacophore models has demonstrated their value in predicting novel actives from collections of FDA approved drugs ^22– 27^. Candidate predicted actives may be assessed by their Fit Value to the pharmacophore model. This score can be used to prioritize compounds for eventual testing. In the current study it was hypothesized that two different classes of compounds showing activity against EBOV *in vitro* and *in vivo* may share a common pharmacophore. Construction of this pharmacophore ( Figure 1) indicated four hydrophobic features and a hydrogen bond acceptor feature. This pharmacophore (with an added van der Waals surface to limit the number of hits retrieved) was then used to screen and score other FDA drugs from a small database and identified 120 and 124 structures for future evaluation *in vitro* testing ( Supplemental Table 2 and Supplemental Table 3). Out of these compounds estradiol and dibucaine had been previously described as active in *in vitro* EBOV assays. This suggested the pharmacophore could retrieve some structurally diverse classes of known hits ^8^.

Recently identified co-crystal structures of the EBOV VP35 protein were used to derive receptor-ligand pharmacophores. These nine receptor-ligand pharmacophores suggested the importance of hydrophobic, hydrogen bonding and negative ionizable interactions to interfere with this protein-protein interaction ( Table 1). Eight out of nine of the pharmacophores had one or more hydrogen bond acceptor feature. These pharmacophores are grossly similar to our ligand based pharmacophore (derived from four FDA approved drugs that inhibit EBOV), as both types of model had multiple hydrophobic features and at least one hydrogen bond acceptor. When we docked the antimalarials and SERMs into a representative VP35 structure these compounds were found to overlap with the X-ray ligand to differing extents. Amodiaquine and chloroquine had LibDock scores greater than 90 and higher than that for the redocked X-ray ligand. This indicated that VP35 may be a potential target for these two distinct classes of compounds. However, it is important to point out that we have not compared docking to other proteins in EBOV and it could also be possible that these molecules are active elsewhere as well as via other mechanisms than by specific binding to proteins ^28, 29^. Further, VP35 may be a preferred target for the antimalarials while the SERMs are not predicted to bind as well as the X-ray ligand. The use of other docking and scoring methods may produce differences in the pose and predicted binding affinity, which could be of interest for further studies.

A combination of the promising efficacy of chloroquine (EC _50_ 16 μM ^8^) and amodiaquine (EC _50_ 8.4 μM ^8^) versus EBOV, their availability and likely low cost should prioritize their further laboratory exploration. Mechanistic studies against VP35 and possibly other proteins should also be pursued and may be enlightened by the observation that both of these compounds also have reported activity against other viruses. For example, chloroquine is active against human coronavirus OC43 ( *in vitro* and in infected mice) as well as SARS ( *in vitro*) ^10, 30, 31^, while amodiaquine also inhibits dengue virus 2 replication and infectivity *in vitro* ^11^.

## Conclusions

In summary, this study has built on the previous publications that identified four FDA approved compounds active against different strains of EBOV ^8, 9^. Our pharmacophore model for SERMs and aminoquinolines suggests that these compounds share multiple chemical features based on their overlap to the four hydrophobic features and a hydrogen bond acceptor ( Figure 1E) and they may have a common mechanism or target. We suggest that VP35 may be the likely target based on the overlap of receptor-based pharmacophores and docking into the crystal structure. Amodiaquine and chloroquine score particularly well in terms of docking to VP35. If this is the case it could provide a means to follow up with other small molecule analogs and/or additional FDA approved drugs that could target this protein-protein interaction. As with our other tuberculosis-focused research ^32, 33^, and computational approaches to repositioning compounds ^34^ we embrace the essentiality for computational predictions to be interrogated through rigorous experimental studies. For example at least two *in silico* docking studies screened commercially available compounds ^14, 15^. We propose that docking FDA approved drugs could also be a viable first step to identifying potential compounds that could be used. We are actively seeking collaborators with experience with EBOV assays to enable further translational studies. We believe this computationally inspired approach may be applicable for other known infectious pathogens that do not have current treatments such as other viruses related to Ebola. Ultimately we need to be able to leverage such approaches to provide antivirals for future pathogens.

## Data availability

F1000Research: Dataset 1. Pharmacophores, receptor ligand models and docking data for FDA-approved drugs inhibiting the Ebola virus, 10.5256/f1000research.5741.d38449 ^35^.

The ligand-based pharmacophore was previously made available: http://figshare.com/articles/Ebola_active_cpds_pharmacophore/1190902.

The following PDB structures were used in this study ( 4IBB, 4IBC, 4IBD, 4IBE, 4IBF, 4IBG, 4IBI, 4IBJ, 4IBK).

For models and advice please contact Sean Ekins ( ekinssean@yahoo.com).

## Figures and Tables

**Supplemental Table 1.** Information for common features pharmacophore generation.

	Maximum Omitted features	Principal
chloroquine	0	1
amodiaquine	0	2
clomiphene	0	1
Toremifene	0	2

**Figure S2.**
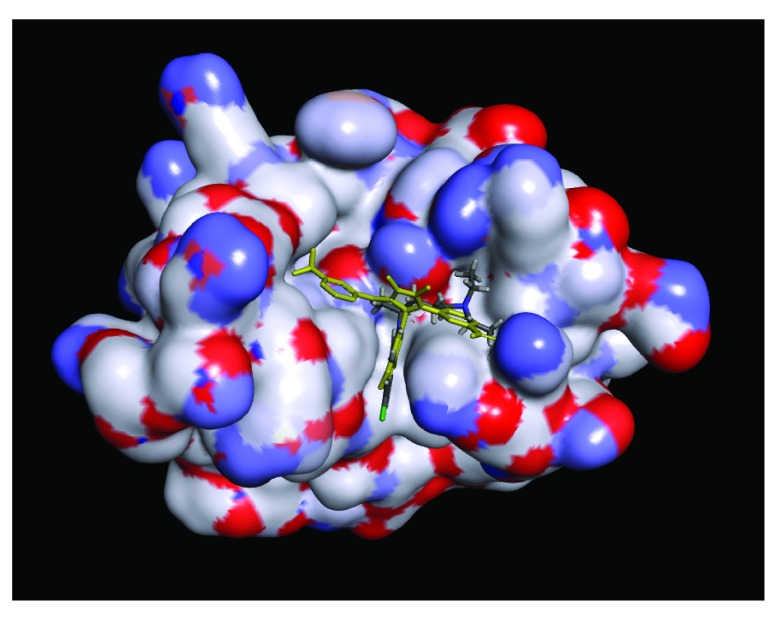
Docking chloroquine in 4IBI showing complete protein. 4IBI ligand VPL57 shown in yellow. Chloroquine (grey). The compounds bind in the first basic patch of the interferon inhibitory domain which is important for the viral nucleoprotein interaction and replication complex formation ^15^.

## Supplementary materials

**Supplemental Table 2.** FDA drugs and common features pharmacophore. The dataset of 2643 molecules was downloaded from the CDD Public Access ( https://www.collaborativedrug.com/pages/public_access) as an sdf and then a 3D database was created in Discovery Studio using FAST conformer generation with up to 255 conformations. The database was searched with the common feature pharmacophore developed from amodiaquine, chloroquine, clomiphene and toremifene. The search 3D database protocol was used with the Fast search method. In some cases the indication for the molecules is not described (ND).

**Supplemental Table 3.** Microsource Spectrum and common features pharmacophore. The dataset of 2311 molecules was provided by Microsource ( http://www.msdiscovery.com/spectrum.html) as an sdf and then a 3D database was created in Discovery Studio using FAST conformer generation with up to 255 conformations. The database was searched with the common feature pharmacophore developed from amodiaquine, chloroquine, clomiphene and toremifene. The search 3D database protocol was used with the Fast search method.
