# Supplementary material for: A common feature pharmacophore for FDA-approved drugs inhibiting the Ebola virus
Source: F1000Res. 2014 Dec 12;3:277. Originally published 2014 Nov 14. [Version 2] doi: 10.12688/f1000research.5741.2 (PMC4304229; doi:10.12688/f1000research.5741.2)
Supplement: Peer review history

# Peer review: Referee response for version 1

**DOI:** 10.5256/f1000research.6137.r6708

**Published:** 2014-12-02

**Authors:** Sandeep Chakraborty

**Affiliations:** 1 Plant Sciences Department, University of California, Davis, CA, USA

Ekins *et al.* have presented a crisp and lucid manuscript on a very relevant topic. They have suggested a methodology to extract common features from four approved compounds that have recently been found to work against the Ebola virus (amodiaquine, chloroquine, clomiphene and toremifene), and define a pharma-

cophore, which has been used to search databases, and identify further compounds for *in vitro* and *in vivo* testing. The *in silico* methodology described here provides an excellent method to quickly screen known compounds for possible therapies against Ebola in particular, and other viruses in general.

Some minor comments.

1. The result that SERMs show lower scores for binding to VP35 is rationalized by the finding that ‘clomiphene and toremifene inhibit EBOV VLP entry with some specificity to GP’ ^1^, and therefore does not probably inhibit VP35.
2. ‘Chloroquine to date has not progressed beyond the mouse EBOV model used in these studies.’ This statement is not clear, does it mean that the others have progressed beyond the mouse EBOV model?
3. The first few compounds in Supplemental Table 2 should be part of the main manuscript as a table.
4. Color coding of pharmacophore features should be in Fig 1 too (it comes earlier than Table 1, where it is described).
5. The structures look better with a white background.
6. A 3 Å RMSD for redocking a given ligand is quite high ^2^. The authors should consider the use of other docking methods, as a comparison.
7. A table of Libdock scores would help easily analyze results (with a mention of whether a higher score is better, and the significance of a score).

The major concern with the manuscript is the use of proprietary software, and data formats, in the study, which makes it difficult for users to probe the resultant docked structures. Further, non-standard formats are subject to the existence of the company which uses it, and not a given in the future.

I have read this submission. I believe that I have an appropriate level of expertise to confirm that it is of an acceptable scientific standard.

## References

[1] JohansenLMBrannanJMDelosSEShoemakerCJStosselALearCHoffstromBGDewaldLESchornbergKLScullyCLehárJHensleyLEWhiteJMOlingerGG: FDA-approved selective estrogen receptor modulators inhibit Ebola virus infection.*Sci Transl Med.*.2013;5(190) : 277190ra7910.1126/scitranslmed.300547123785035PMC3955358

[2] SándorMKissRKeseruGM: Virtual fragment docking by Glide: a validation study on 190 protein-fragment complexes.*J Chem Inf Model.*.2010;50(6) : 2771165-117210.1021/ci1000407Reference source20459088

# Response

**Published:** 2014-12-09

**Authors:** Sean Ekins

**Affiliations:** Collaborations in Chemistry, USA

*Ekins et al. have presented a crisp and lucid manuscript on a very relevant topic. They have suggested a methodology to extract common features from four approved compounds that have recently been found to work against the Ebola virus (amodiaquine, chloroquine, clomiphene and toremifene), and define a pharma-*

*cophore, which has been used to search databases, and identify further compounds for in vitro and in vivo testing. The in silico methodology described here provides an excellent method to quickly screen known compounds for possible therapies against Ebola in particular, and other viruses in general.*

**Response:** Thank you for your constructive comments.

*Some minor comments.*

1. *The result that SERMs show lower scores for binding to VP35 is rationalized by the finding that ‘clomiphene and toremifene inhibit EBOV VLP entry with some specificity to GP’ ^1^, and therefore does not probably inhibit VP35.*
**Response:** While the lower docking scores are noted I do not think this necessarily excludes them from inhibiting, as we know docking scores may not be that accurate and in this case, docking was used to answer the question could they fit. It’s pretty clear that a wide variety of drugs could fit based on the binding site size and accessibility.
2. *‘Chloroquine to date has not progressed beyond the mouse EBOV model used in these studies.’ This statement is not clear, does it mean that the others have progressed beyond the mouse EBOV model?*
**Response:** From discussions with the author on the paper that described Chloroquine as active versus EBOV *in vitro* and in mouse, this work has not gone beyond the mouse model of EBOV.
3. *The first few compounds in Supplemental Table 2 should be part of the main manuscript as a table.*
**Response:** Because this data is available easily on the website, I do not see any benefits of taking these compounds out of this supplemental table and putting them into the body of the manuscript. It might also add more confusion cutting the table up.
4. *Color coding of pharmacophore features should be in Fig 1 too (it comes earlier than Table 1, where it is described).*
**Response:** Thank you – this has now been added.
5. *The structures look better with a white background.*
**Response:** I think this is a personal preference, the structures are clear in our opinion with a black background. I have not had this suggestion previously with other publications regarding the background color.
6. *A 3 Å RMSD for redocking a given ligand is quite high ^2^. The authors should consider the use of other docking methods, as a comparison.*
**Response**: The study was not intended as an exhaustive docking comparison, there are plenty of these in the literature as noted by the reviewer. I agreed the redocking RMSD was high, but I also provided some justification for the result (difficulty of predicting orientations for compounds binding in what is a relatively hydrophobic and shallow pocket). If others want to use different methods and perform a comparison for this target I would be supportive. 
7. *A table of Libdock scores would help easily analyze results (with a mention of whether a higher score is better, and the significance of a score).*
**Response:** The Libdock scores for the best poses are in the ‘4IBI Libdock docking data best poses” file. A higher score is better and this has been added to the results section

*The major concern with the manuscript is the use of proprietary software, and data formats, in the study, which makes it difficult for users to probe the resultant docked structures. Further, non-standard formats are subject to the existence of the company which uses it, and not a given in the future.*

**Response:** All of the models were generated with the proprietary software Discovery studio, and all files have been provided. The comment about such software is true, while I support using open software, I have yet to find an open source pharmacophore tool as good as that in Discovery Studio to date. It is also more convenient to use this software generating pharmacophores, receptor-ligand pharmacophores and docking in the same place. The types of analysis I have described could be repeated with any software, open source or proprietary. My hope is that by making this work openly accessible others will be inspired to pursue computational approaches with EBOV. Perhaps the community could propose the use of standards for open pharmacophore files as well. By publishing in this journal we are making all our data open even though they are in proprietary formats, I do not think this should preclude publication.

# Peer review: Referee response for version 1

**DOI:** 10.5256/f1000research.6137.r6707

**Published:** 2014-12-01

**Authors:** Gloria Fuentes

**Affiliations:** 1 Center for Life Sciences Technologies, RIKEN, Saitama, Japan

The current Ebola crisis in West Africa has shattered all expectations by continuing to grow months following the initial case. This has stimulated a massive and global emergency response, and it has challenged the health protocols and by extension, the efforts of scientific community.

The authors have carried out a computational analysis using several compounds detected in two previous high throughput screens to build a pharmacophore model. The key features of such a model are used to scan databases of small molecules. They have come up with a list of putative inhibitors. Their study will have a stronger scientific impact if the authors could elaborate more in suggestions on how the best ranked compounds will increase the binding affinity, based in the structural model VP30-inhibitors that they have built. 

In parallel, they observed a highly overlapping between the motifs in the pharmacophore and those found in the crystal structures of several inhibitors of the viral protein 35. Based on this fact, and in their results in an in-silico docking, they propose that the most likely inhibitory mechanism for these compounds is the targeting of the protein-protein interaction involving this protein. 

In this regard, the authors should extend their study to include different docking protocols, including different programs, in an attempt to verify their results. As they mention in the text (page 4), the redocking of the ligand in 4IBI to the protein does not show the crystal structure binding mode accurately. These different settings could help in a better prediction of the ligand orientations.

Concerning the docking and proposed mechanism, I wonder how different the other solved nucleocapsid proteins are from a structural and sequence point of view, in order to make the authors point that VP35 is indeed the target. Would it be possible to explore for surface patches with similar physico-chemical features? In the case of VP30, a potential binding pocket for small-molecule inhibitors has been suggested by Hartlieb *et al. *(2007).  How good or bad the overlapping with the built pharmachophore is for this case?

To complete the structural understanding of the action of these compounds, a figure displaying their location on the protein surface as well as the binding site for RNA would clarify their role in the inhibition of protein-protein interactions.  

In summary, the manuscript describes an interesting and fast approach to identify putative inhibitors for a currently serious target as Ebola virus. Although their results should be experimental validated to confirm their finding, this computational study and further extensions of it are of the great value.

I have read this submission. I believe that I have an appropriate level of expertise to confirm that it is of an acceptable scientific standard.

# Response

**Published:** 2014-12-09

**Authors:** Sean Ekins

**Affiliations:** Collaborations in Chemistry, USA

- *The current Ebola crisis in West Africa has shattered all expectations by continuing to grow months following the initial case. This has stimulated a massive and global emergency response, and it has challenged the health protocols and by extension, the efforts of scientific community.*
*The authors have carried out a computational analysis using several compounds detected in two previous high throughput screens to build a pharmacophore model. The key features of such a model are used to scan databases of small molecules. They have come up with a list of putative inhibitors. Their study will have a stronger scientific impact if the authors could elaborate more in suggestions on how the best ranked compounds will increase the binding affinity, based in the structural model VP30-inhibitors that they have built.*
**Response**: To clarify, we have focused on VP35 not VP30. I am not aware of an X-ray structure with ligand bound for VP30. The same type of approach could certainly be pursued with other EBOV targets. We produced a common features pharmacophore for the 4 compounds, and after looking at the VP35 receptor-ligand pharmacophores proposed that there may be some overlap, and then this led to docking the 4 compounds in the X-ray structures. Our intent was not to design molecules but to use the available methods to perhaps infer a potential target/mechanism and then perhaps researchers would want to test the compounds. We do not have access to experimentally test these predictions, but this manuscript may lead to others doing this work perhaps. Whether one wants to use the Libdock score for (absolute) prediction of binding affinity interactions is debatable, rather this approach might help to limit or prioritize which compounds to test. 
- *In parallel, they observed a highly overlapping between the motifs in the pharmacophore and those found in the crystal structures of several inhibitors of the viral protein 35. Based on this fact, and in their results in an in-silico docking, they propose that the most likely inhibitory mechanism for these compounds is the targeting of the protein-protein interaction involving this protein. *
**Response: **VP35 may be a target for these compounds although we do not discount other targets or non-target related mechanisms.
- *In this regard, the authors should extend their study to include different docking protocols, including different programs, in an attempt to verify their results. As they mention in the text (page 4), the redocking of the ligand in 4IBI to the protein does not show the crystal structure binding mode accurately. These different settings could help in a better prediction of the ligand orientations.*
**Response**: As explained earlier, our study is not intended to be an exhaustive evaluation of docking tools, we have used different computational approaches to suggest that the FDA drugs may have a common pharmacophore, which seems to be similar to that of the ligands co-crystallized with VP-35. Finally docking suggests they may fit into the pocket that the co-crystal ligands bind to. The work proposes that the compounds could fit in the binding site, but it is unclear what additional value more docking would add unless we were going to try to predict and then generate the X-ray structure of these FDA drugs. Certainly if experts in docking or crystallography want to pursue this target they can.
- *Concerning the docking and proposed mechanism, I wonder how different the other solved nucleocapsid proteins are from a structural and sequence point of view, in order to make the authors point that VP35 is indeed the target. Would it be possible to explore for surface patches with similar physico-chemical features? In the case of VP30, a potential binding pocket for small-molecule inhibitors has been suggested by Hartlieb et al. (2007).  How good or bad the overlapping with the built pharmachophore is for this case?*
**Response:** this is indeed a very good point. We are not experts on these proteins. I think the proposed work could be done, the difficulty may be that there is no crystal structure (that I can see) with a ligand bound that would be a useful guide to binding in this pocket and would be essential for a receptor-ligand pharmacophore to be built.
- *To complete the structural understanding of the action of these compounds, a figure displaying their location on the protein surface as well as the binding site for RNA would clarify their role in the inhibition of protein-protein interactions.*
**Response:** we have now added Figure S2 which shows the molecules in the context of the full protein. They are in the site suggested in ref 15 as important for the nucleoprotein interaction.
- *In summary, the manuscript describes an interesting and fast approach to identify putative inhibitors for a currently serious target as Ebola virus. Although their results should be experimental validated to confirm their finding, this computational study and further extensions of it are of the great value.*
**Response:** Thank you for your suggestions, we agree and would encourage other scientists to test whether these compounds are targeting VP35 or VP 30 as you propose, or having an alternative mechanism. I think we would also be happy to see any of these molecules progress into other animal models of EBOV.
